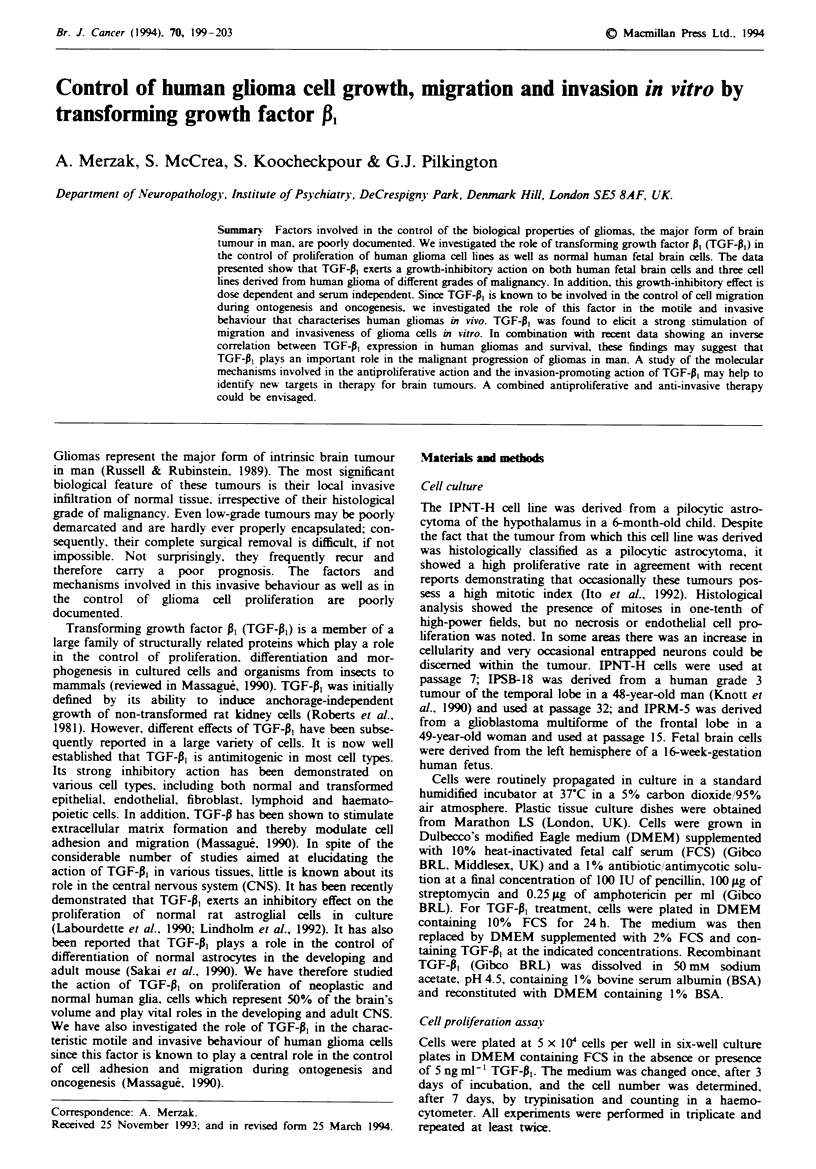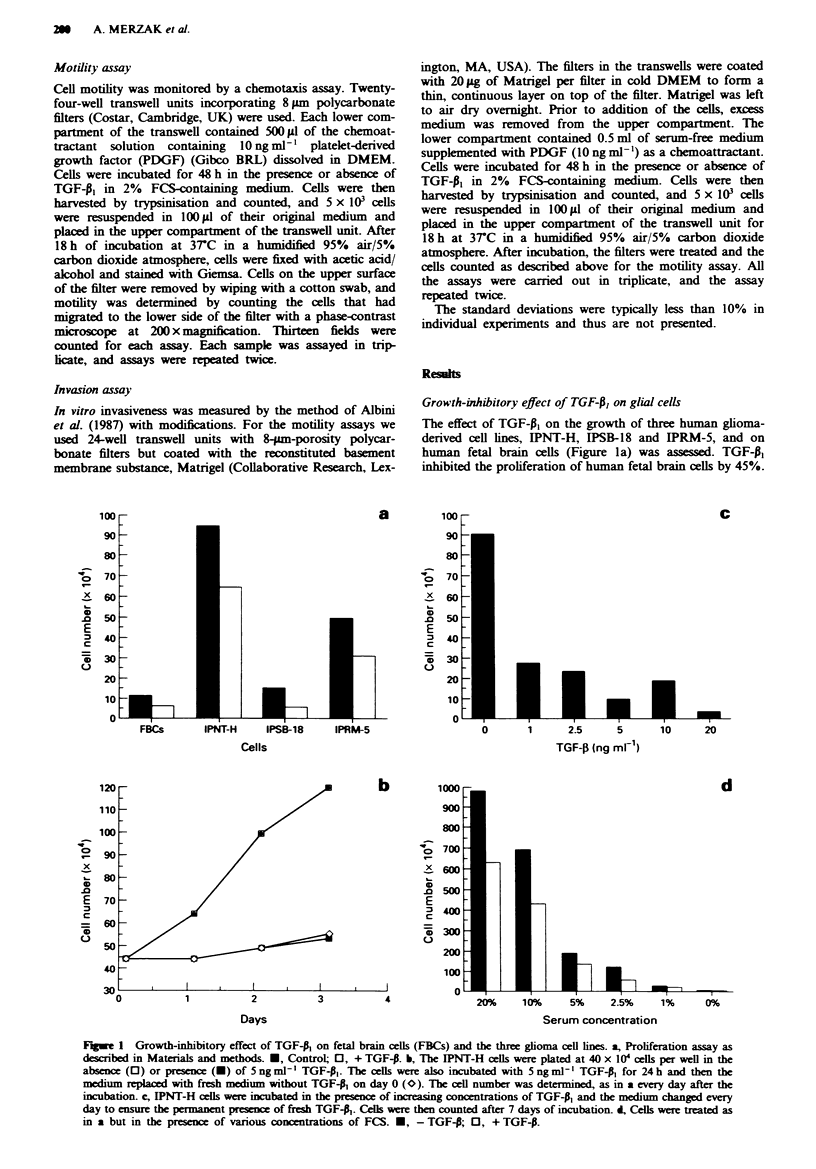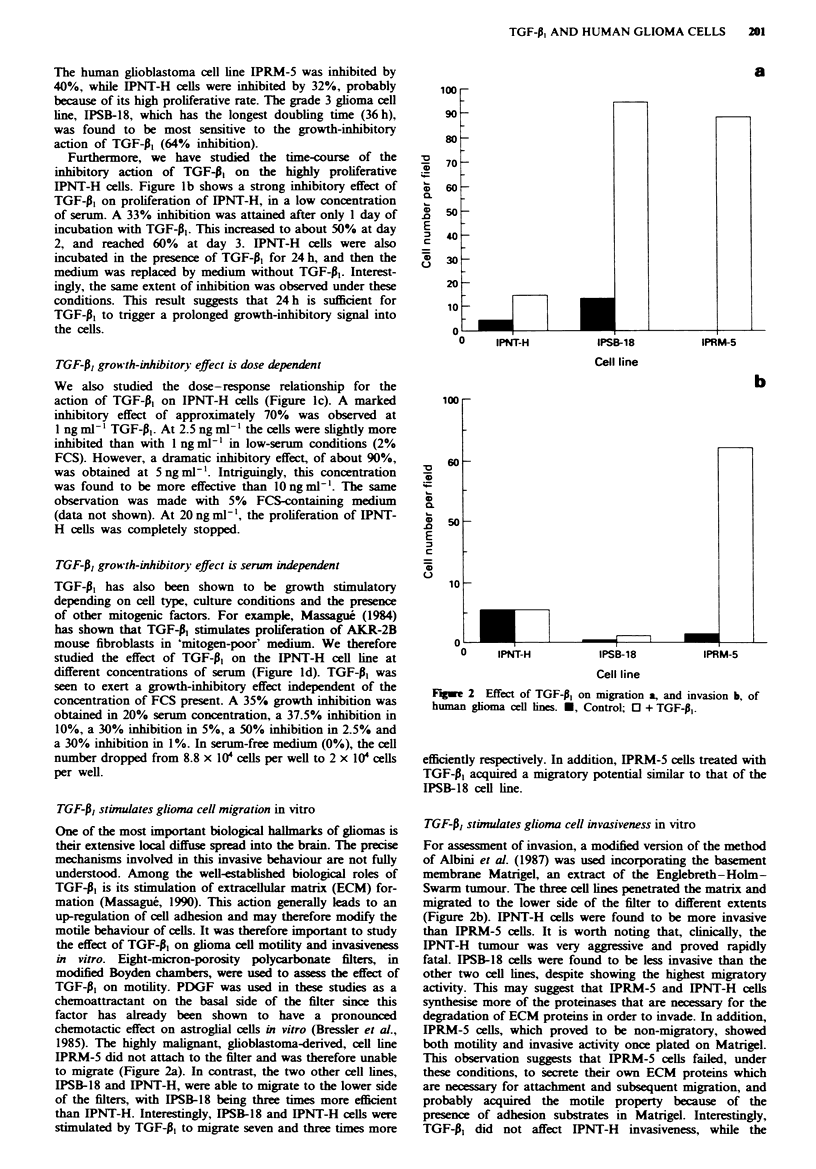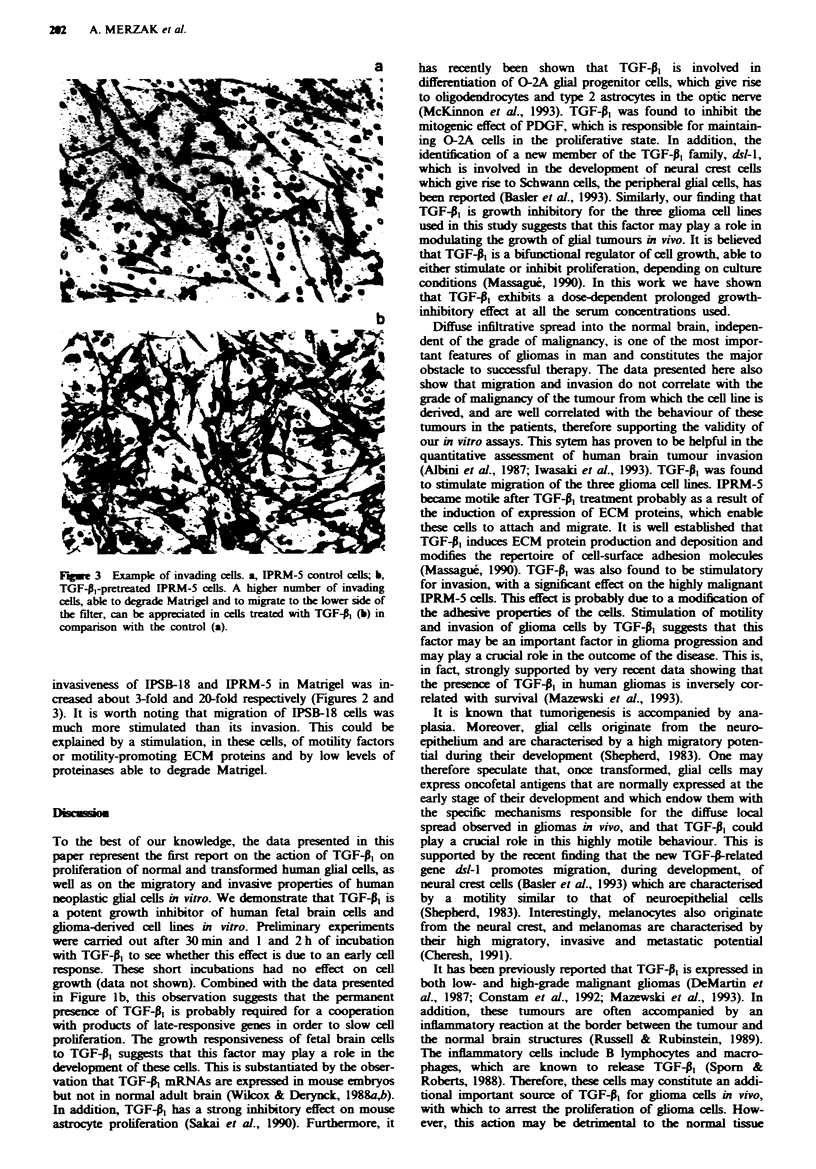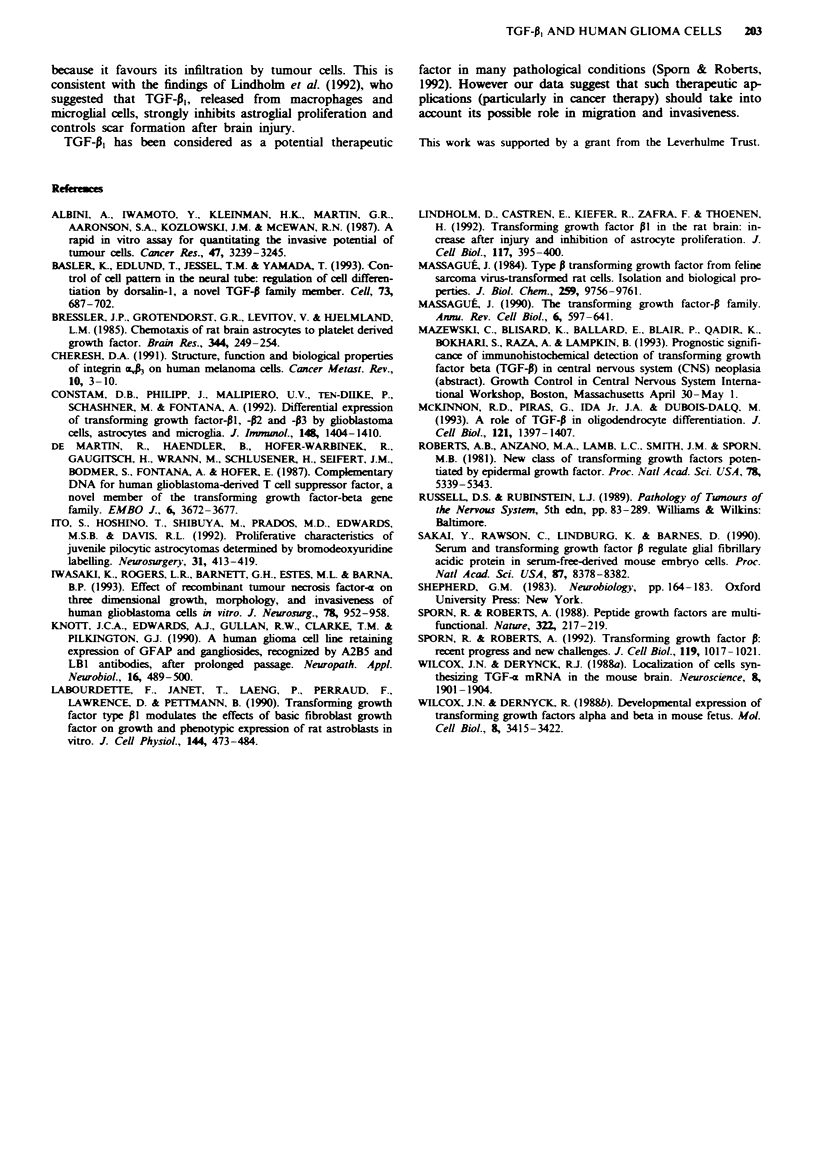# Control of human glioma cell growth, migration and invasion in vitro by transforming growth factor beta 1.

**DOI:** 10.1038/bjc.1994.280

**Published:** 1994-08

**Authors:** A. Merzak, S. McCrea, S. Koocheckpour, G. J. Pilkington

**Affiliations:** Department of Neuropathology, Institute of Psychiatry, London, UK.

## Abstract

**Images:**


Br. J. Cancer (1994), 70, 199-203 © Macmillan Press Ltd., 1994

### Control of human glioma cell growth, migration and invasion *in vitro* by transforming growth factor $\beta_1$

A. Merzak, S. McCrea, S. Koocheckpour & G.J. Pilkington

*Department of Neuropathology, Institute of Psychiatry, DeCrespigny Park, Denmark Hill, London SE5 8AF, UK.*

**Summary** Factors involved in the control of the biological properties of gliomas, the major form of brain tumour in man, are poorly documented. We investigated the role of transforming growth factor $\beta_1$ (TGF-$\beta_1$) in the control of proliferation of human glioma cell lines as well as normal human fetal brain cells. The data presented show that TGF-$\beta_1$ exerts a growth-inhibitory action on both human fetal brain cells and three cell lines derived from human glioma of different grades of malignancy. In addition, this growth-inhibitory effect is dose dependent and serum independent. Since TGF-$\beta_1$ is known to be involved in the control of cell migration during ontogenesis and oncogenesis, we investigated the role of this factor in the motile and invasive behaviour that characterises human gliomas *in vivo*. TGF-$\beta_1$ was found to elicit a strong stimulation of migration and invasiveness of glioma cells *in vitro*. In combination with recent data showing an inverse correlation between TGF-$\beta_1$ expression in human gliomas and survival, these findings may suggest that TGF-$\beta_1$ plays an important role in the malignant progression of gliomas in man. A study of the molecular mechanisms involved in the antiproliferative action and the invasion-promoting action of TGF-$\beta_1$ may help to identify new targets in therapy for brain tumours. A combined antiproliferative and anti-invasive therapy could be envisaged.

Gliomas represent the major form of intrinsic brain tumour in man (Russell & Rubinstein, 1989). The most significant biological feature of these tumours is their local invasive infiltration of normal tissue, irrespective of their histological grade of malignancy. Even low-grade tumours may be poorly demarcated and are hardly ever properly encapsulated; consequently, their complete surgical removal is difficult, if not impossible. Not surprisingly, they frequently recur and therefore carry a poor prognosis. The factors and mechanisms involved in this invasive behaviour as well as in the control of glioma cell proliferation are poorly documented.

Transforming growth factor $\beta_1$ (TGF-$\beta_1$) is a member of a large family of structurally related proteins which play a role in the control of proliferation, differentiation and morphogenesis in cultured cells and organisms from insects to mammals (reviewed in Massagué, 1990). TGF-$\beta_1$ was initially defined by its ability to induce anchorage-independent growth of non-transformed rat kidney cells (Roberts *et al.*, 1981). However, different effects of TGF-$\beta_1$ have been subsequently reported in a large variety of cells. It is now well established that TGF-$\beta_1$ is antimitogenic in most cell types. Its strong inhibitory action has been demonstrated on various cell types, including both normal and transformed epithelial, endothelial, fibroblast, lymphoid and haematopoietic cells. In addition, TGF-$\beta$ has been shown to stimulate extracellular matrix formation and thereby modulate cell adhesion and migration (Massagué, 1990). In spite of the considerable number of studies aimed at elucidating the action of TGF-$\beta_1$ in various tissues, little is known about its role in the central nervous system (CNS). It has been recently demonstrated that TGF-$\beta_1$ exerts an inhibitory effect on the proliferation of normal rat astroglial cells in culture (Labourdette *et al.*, 1990; Lindholm *et al.*, 1992). It has also been reported that TGF-$\beta_1$ plays a role in the control of differentiation of normal astrocytes in the developing and adult mouse (Sakai *et al.*, 1990). We have therefore studied the action of TGF-$\beta_1$ on proliferation of neoplastic and normal human glia, cells which represent 50% of the brain's volume and play vital roles in the developing and adult CNS. We have also investigated the role of TGF-$\beta_1$ in the characteristic motile and invasive behaviour of human glioma cells since this factor is known to play a central role in the control of cell adhesion and migration during ontogenesis and oncogenesis (Massagué, 1990).

Correspondence: A. Merzak.
Received 25 November 1993; and in revised form 25 March 1994.

### Materials and methods

### *Cell culture*

The IPNT-H cell line was derived from a pilocytic astrocytoma of the hypothalamus in a 6-month-old child. Despite the fact that the tumour from which this cell line was derived was histologically classified as a pilocytic astrocytoma, it showed a high proliferative rate in agreement with recent reports demonstrating that occasionally these tumours possess a high mitotic index (Ito *et al.*, 1992). Histological analysis showed the presence of mitoses in one-tenth of high-power fields, but no necrosis or endothelial cell proliferation was noted. In some areas there was an increase in cellularity and very occasional entrapped neurons could be discerned within the tumour. IPNT-H cells were used at passage 7; IPSB-18 was derived from a human grade 3 tumour of the temporal lobe in a 48-year-old man (Knott *et al.*, 1990) and used at passage 32; and IPRM-5 was derived from a glioblastoma multiforme of the frontal lobe in a 49-year-old woman and used at passage 15. Fetal brain cells were derived from the left hemisphere of a 16-week-gestation human fetus.

Cells were routinely propagated in culture in a standard humidified incubator at 37°C in a 5% carbon dioxide/95% air atmosphere. Plastic tissue culture dishes were obtained from Marathon LS (London, UK). Cells were grown in Dulbecco's modified Eagle medium (DMEM) supplemented with 10% heat-inactivated fetal calf serum (FCS) (Gibco BRL, Middlesex, UK) and a 1% antibiotic/antimycotic solution at a final concentration of 100 IU of pencillin, 100 μg of streptomycin and 0.25 μg of amphotericin per ml (Gibco BRL). For TGF-$\beta_1$ treatment, cells were plated in DMEM containing 10% FCS for 24 h. The medium was then replaced by DMEM supplemented with 2% FCS and containing TGF-$\beta_1$ at the indicated concentrations. Recombinant TGF-$\beta_1$ (Gibco BRL) was dissolved in 50 mM sodium acetate, pH 4.5, containing 1% bovine serum albumin (BSA) and reconstituted with DMEM containing 1% BSA.

### *Cell proliferation assay*

Cells were plated at $5 \times 10^4$ cells per well in six-well culture plates in DMEM containing FCS in the absence or presence of 5 ng $ml^{-1}$ TGF-$\beta_1$. The medium was changed once, after 3 days of incubation, and the cell number was determined, after 7 days, by trypinisation and counting in a haemocytometer. All experiments were performed in triplicate and repeated at least twice.

### *Motility assay*

Cell motility was monitored by a chemotaxis assay. Twenty-four-well transwell units incorporating 8 μm polycarbonate filters (Costar, Cambridge, UK) were used. Each lower compartment of the transwell contained 500 μl of the chemoattractant solution containing 10 ng $ml^{-1}$ platelet-derived growth factor (PDGF) (Gibco BRL) dissolved in DMEM. Cells were incubated for 48 h in the presence or absence of TGF-$\beta_1$ in 2% FCS-containing medium. Cells were then harvested by trypsinisation and counted, and $5 \times 10^3$ cells were resuspended in 100 μl of their original medium and placed in the upper compartment of the transwell unit. After 18 h of incubation at 37°C in a humidified 95% air/5% carbon dioxide atmosphere, cells were fixed with acetic acid/alcohol and stained with Giemsa. Cells on the upper surface of the filter were removed by wiping with a cotton swab, and motility was determined by counting the cells that had migrated to the lower side of the filter with a phase-contrast microscope at 200 × magnification. Thirteen fields were counted for each assay. Each sample was assayed in triplicate, and assays were repeated twice.

### *Invasion assay*

*In vitro* invasiveness was measured by the method of Albini *et al.* (1987) with modifications. For the motility assays we used 24-well transwell units with 8-μm-porosity polycarbonate filters but coated with the reconstituted basement membrane substance, Matrigel (Collaborative Research, Lexington, MA, USA). The filters in the transwells were coated with 20 μg of Matrigel per filter in cold DMEM to form a thin, continuous layer on top of the filter. Matrigel was left to air dry overnight. Prior to addition of the cells, excess medium was removed from the upper compartment. The lower compartment contained 0.5 ml of serum-free medium supplemented with PDGF (10 ng $ml^{-1}$) as a chemoattractant. Cells were incubated for 48 h in the presence or absence of TGF-$\beta_1$ in 2% FCS-containing medium. Cells were then harvested by trypsinisation and counted, and $5 \times 10^3$ cells were resuspended in 100 μl of their original medium and placed in the upper compartment of the transwell unit for 18 h at 37°C in a humidified 95% air/5% carbon dioxide atmosphere. After incubation, the filters were treated and the cells counted as described above for the motility assay. All the assays were carried out in triplicate, and the assay repeated twice.

The standard deviations were typically less than 10% in individual experiments and thus are not presented.

### Results

### *Growth-inhibitory effect of TGF-$\beta_1$ on glial cells*

The effect of TGF-$\beta_1$ on the growth of three human glioma-derived cell lines, IPNT-H, IPSB-18 and IPRM-5, and on human fetal brain cells (Figure 1a) was assessed. TGF-$\beta_1$ inhibited the proliferation of human fetal brain cells by 45%.

**Figure 1** Growth-inhibitory effect of TGF-$\beta_1$ on fetal brain cells (FBCs) and the three glioma cell lines. **a**, Proliferation assay as described in Materials and methods. ■, Control; □, + TGF-β. **b**, The IPNT-H cells were plated at $40 \times 10^4$ cells per well in the absence (□) or presence (■) of 5 ng $ml^{-1}$ TGF-$\beta_1$. The cells were also incubated with 5 ng $ml^{-1}$ TGF-$\beta_1$ for 24 h and then the medium replaced with fresh medium without TGF-$\beta_1$ on day 0 (◇). The cell number was determined, as in **a** every day after the incubation. **c**, IPNT-H cells were incubated in the presence of increasing concentrations of TGF-$\beta_1$ and the medium changed every day to ensure the permanent presence of fresh TGF-$\beta_1$. Cells were then counted after 7 days of incubation. **d**, Cells were treated as in **a** but in the presence of various concentrations of FCS. ■, − TGF-β; □, + TGF-β.

The human glioblastoma cell line IPRM-5 was inhibited by 40%, while IPNT-H cells were inhibited by 32%, probably because of its high proliferative rate. The grade 3 glioma cell line, IPSB-18, which has the longest doubling time (36 h), was found to be most sensitive to the growth-inhibitory action of TGF-$\beta_1$ (64% inhibition).

Furthermore, we have studied the time-course of the inhibitory action of TGF-$\beta_1$ on the highly proliferative IPNT-H cells. Figure 1b shows a strong inhibitory effect of TGF-$\beta_1$ on proliferation of IPNT-H, in a low concentration of serum. A 33% inhibition was attained after only 1 day of incubation with TGF-$\beta_1$. This increased to about 50% at day 2, and reached 60% at day 3. IPNT-H cells were also incubated in the presence of TGF-$\beta_1$ for 24 h, and then the medium was replaced by medium without TGF-$\beta_1$. Interestingly, the same extent of inhibition was observed under these conditions. This result suggests that 24 h is sufficient for TGF-$\beta_1$ to trigger a prolonged growth-inhibitory signal into the cells.

*TGF-$\beta_1$ growth-inhibitory effect is dose dependent*

We also studied the dose-response relationship for the action of TGF-$\beta_1$ on IPNT-H cells (Figure 1c). A marked inhibitory effect of approximately 70% was observed at 1 ng $ml^{-1}$ TGF-$\beta_1$. At 2.5 ng $ml^{-1}$ the cells were slightly more inhibited than with 1 ng $ml^{-1}$ in low-serum conditions (2% FCS). However, a dramatic inhibitory effect, of about 90%, was obtained at 5 ng $ml^{-1}$. Intriguingly, this concentration was found to be more effective than 10 ng $ml^{-1}$. The same observation was made with 5% FCS-containing medium (data not shown). At 20 ng $ml^{-1}$, the proliferation of IPNT-H cells was completely stopped.

*TGF-$\beta_1$ growth-inhibitory effect is serum independent*

TGF-$\beta_1$ has also been shown to be growth stimulatory depending on cell type, culture conditions and the presence of other mitogenic factors. For example, Massagué (1984) has shown that TGF-$\beta_1$ stimulates proliferation of AKR-2B mouse fibroblasts in 'mitogen-poor' medium. We therefore studied the effect of TGF-$\beta_1$ on the IPNT-H cell line at different concentrations of serum (Figure 1d). TGF-$\beta_1$ was seen to exert a growth-inhibitory effect independent of the concentration of FCS present. A 35% growth inhibition was obtained in 20% serum concentration, a 37.5% inhibition in 10%, a 30% inhibition in 5%, a 50% inhibition in 2.5% and a 30% inhibition in 1%. In serum-free medium (0%), the cell number dropped from $8.8 \times 10^4$ cells per well to $2 \times 10^4$ cells per well.

*TGF-$\beta_1$ stimulates glioma cell migration* in vitro

One of the most important biological hallmarks of gliomas is their extensive local diffuse spread into the brain. The precise mechanisms involved in this invasive behaviour are not fully understood. Among the well-established biological roles of TGF-$\beta_1$ is its stimulation of extracellular matrix (ECM) formation (Massagué, 1990). This action generally leads to an up-regulation of cell adhesion and may therefore modify the motile behaviour of cells. It was therefore important to study the effect of TGF-$\beta_1$ on glioma cell motility and invasiveness *in vitro*. Eight-micron-porosity polycarbonate filters, in modified Boyden chambers, were used to assess the effect of TGF-$\beta_1$ on motility. PDGF was used in these studies as a chemoattractant on the basal side of the filter since this factor has already been shown to have a pronounced chemotactic effect on astroglial cells *in vitro* (Bressler *et al.*, 1985). The highly malignant, glioblastoma-derived, cell line IPRM-5 did not attach to the filter and was therefore unable to migrate (Figure 2a). In contrast, the two other cell lines, IPSB-18 and IPNT-H, were able to migrate to the lower side of the filters, with IPSB-18 being three times more efficient than IPNT-H. Interestingly, IPSB-18 and IPNT-H cells were stimulated by TGF-$\beta_1$ to migrate seven and three times more efficiently respectively. In addition, IPRM-5 cells treated with TGF-$\beta_1$ acquired a migratory potential similar to that of the IPSB-18 cell line.

**Figure 2** Effect of TGF-$\beta_1$ on migration **a**, and invasion **b**, of human glioma cell lines. ■, Control; □ + TGF-$\beta_1$.

*TGF-$\beta_1$ stimulates glioma cell invasiveness* in vitro

For assessment of invasion, a modified version of the method of Albini *et al.* (1987) was used incorporating the basement membrane Matrigel, an extract of the Englebreth-Holm-Swarm tumour. The three cell lines penetrated the matrix and migrated to the lower side of the filter to different extents (Figure 2b). IPNT-H cells were found to be more invasive than IPRM-5 cells. It is worth noting that, clinically, the IPNT-H tumour was very aggressive and proved rapidly fatal. IPSB-18 cells were found to be less invasive than the other two cell lines, despite showing the highest migratory activity. This may suggest that IPRM-5 and IPNT-H cells synthesise more of the proteinases that are necessary for the degradation of ECM proteins in order to invade. In addition, IPRM-5 cells, which proved to be non-migratory, showed both motility and invasive activity once plated on Matrigel. This observation suggests that IPRM-5 cells failed, under these conditions, to secrete their own ECM proteins which are necessary for attachment and subsequent migration, and probably acquired the motile property because of the presence of adhesion substrates in Matrigel. Interestingly, TGF-$\beta_1$ did not affect IPNT-H invasiveness, while the

**Figure 3** Example of invading cells. **a**, IPRM-5 control cells; **b**, TGF-$\beta_1$-pretreated IPRM-5 cells. A higher number of invading cells, able to degrade Matrigel and to migrate to the lower side of the filter, can be appreciated in cells treated with TGF-$\beta_1$ (**b**) in comparison with the control (**a**).

invasiveness of IPSB-18 and IPRM-5 in Matrigel was increased about 3-fold and 20-fold respectively (Figures 2 and 3). It is worth noting that migration of IPSB-18 cells was much more stimulated than its invasion. This could be explained by a stimulation, in these cells, of motility factors or motility-promoting ECM proteins and by low levels of proteinases able to degrade Matrigel.

### Discussion

To the best of our knowledge, the data presented in this paper represent the first report on the action of TGF-$\beta_1$ on proliferation of normal and transformed human glial cells, as well as on the migratory and invasive properties of human neoplastic glial cells *in vitro*. We demonstrate that TGF-$\beta_1$ is a potent growth inhibitor of human fetal brain cells and glioma-derived cell lines *in vitro*. Preliminary experiments were carried out after 30 min and 1 and 2 h of incubation with TGF-$\beta_1$ to see whether this effect is due to an early cell response. These short incubations had no effect on cell growth (data not shown). Combined with the data presented in Figure 1b, this observation suggests that the permanent presence of TGF-$\beta_1$ is probably required for a cooperation with products of late-responsive genes in order to slow cell proliferation. The growth responsiveness of fetal brain cells to TGF-$\beta_1$ suggests that this factor may play a role in the development of these cells. This is substantiated by the observation that TGF-$\beta_1$ mRNAs are expressed in mouse embryos but not in normal adult brain (Wilcox & Derynck, 1988*a*,*b*). In addition, TGF-$\beta_1$ has a strong inhibitory effect on mouse astrocyte proliferation (Sakai *et al.*, 1990). Furthermore, it has recently been shown that TGF-$\beta_1$ is involved in differentiation of O-2A glial progenitor cells, which give rise to oligodendrocytes and type 2 astrocytes in the optic nerve (McKinnon *et al.*, 1993). TGF-$\beta_1$ was found to inhibit the mitogenic effect of PDGF, which is responsible for maintaining O-2A cells in the proliferative state. In addition, the identification of a new member of the TGF-$\beta_1$ family, *dsl*-1, which is involved in the development of neural crest cells which give rise to Schwann cells, the peripheral glial cells, has been reported (Basler *et al.*, 1993). Similarly, our finding that TGF-$\beta_1$ is growth inhibitory for the three glioma cell lines used in this study suggests that this factor may play a role in modulating the growth of glial tumours *in vivo*. It is believed that TGF-$\beta_1$ is a bifunctional regulator of cell growth, able to either stimulate or inhibit proliferation, depending on culture conditions (Massagué, 1990). In this work we have shown that TGF-$\beta_1$ exhibits a dose-dependent prolonged growth-inhibitory effect at all the serum concentrations used.

Diffuse infiltrative spread into the normal brain, independent of the grade of malignancy, is one of the most important features of gliomas in man and constitutes the major obstacle to successful therapy. The data presented here also show that migration and invasion do not correlate with the grade of malignancy of the tumour from which the cell line is derived, and are well correlated with the behaviour of these tumours in the patients, therefore supporting the validity of our *in vitro* assays. This sytem has proven to be helpful in the quantitative assessment of human brain tumour invasion (Albini *et al.*, 1987; Iwasaki *et al.*, 1993). TGF-$\beta_1$ was found to stimulate migration of the three glioma cell lines. IPRM-5 became motile after TGF-$\beta_1$ treatment probably as a result of the induction of expression of ECM proteins, which enable these cells to attach and migrate. It is well established that TGF-$\beta_1$ induces ECM protein production and deposition and modifies the repertoire of cell-surface adhesion molecules (Massagué, 1990). TGF-$\beta_1$ was also found to be stimulatory for invasion, with a significant effect on the highly malignant IPRM-5 cells. This effect is probably due to a modification of the adhesive properties of the cells. Stimulation of motility and invasion of glioma cells by TGF-$\beta_1$ suggests that this factor may be an important factor in glioma progression and may play a crucial role in the outcome of the disease. This is, in fact, strongly supported by very recent data showing that the presence of TGF-$\beta_1$ in human gliomas is inversely correlated with survival (Mazewski *et al.*, 1993).

It is known that tumorigenesis is accompanied by anaplasia. Moreover, glial cells originate from the neuroepithelium and are characterised by a high migratory potential during their development (Shepherd, 1983). One may therefore speculate that, once transformed, glial cells may express oncofetal antigens that are normally expressed at the early stage of their development and which endow them with the specific mechanisms responsible for the diffuse local spread observed in gliomas *in vivo*, and that TGF-$\beta_1$ could play a crucial role in this highly motile behaviour. This is supported by the recent finding that the new TGF-$\beta$-related gene *dsl*-1 promotes migration, during development, of neural crest cells (Basler *et al.*, 1993) which are characterised by a motility similar to that of neuroepithelial cells (Shepherd, 1983). Interestingly, melanocytes also originate from the neural crest, and melanomas are characterised by their high migratory, invasive and metastatic potential (Cheresh, 1991).

It has been previously reported that TGF-$\beta_1$ is expressed in both low- and high-grade malignant gliomas (DeMartin *et al.*, 1987; Constam *et al.*, 1992; Mazewski *et al.*, 1993). In addition, these tumours are often accompanied by an inflammatory reaction at the border between the tumour and the normal brain structures (Russell & Rubinstein, 1989). The inflammatory cells include B lymphocytes and macrophages, which are known to release TGF-$\beta_1$ (Sporn & Roberts, 1988). Therefore, these cells may constitute an additional important source of TGF-$\beta_1$ for glioma cells *in vivo*, with which to arrest the proliferation of glioma cells. However, this action may be detrimental to the normal tissue because it favours its infiltration by tumour cells. This is consistent with the findings of Lindholm *et al.* (1992), who suggested that TGF-$\beta_1$, released from macrophages and microglial cells, strongly inhibits astroglial proliferation and controls scar formation after brain injury.

TGF-$\beta_1$ has been considered as a potential therapeutic factor in many pathological conditions (Sporn & Roberts, 1992). However our data suggest that such therapeutic applications (particularly in cancer therapy) should take into account its possible role in migration and invasiveness.

This work was supported by a grant from the Leverhulme Trust.

**References**

ALBINI, A., IWAMOTO, Y., KLEINMAN, H.K., MARTIN, G.R., AARONSON, S.A., KOZLOWSKI, J.M. & MCEWAN, R.N. (1987). A rapid in vitro assay for quantitating the invasive potential of tumour cells. *Cancer Res.*, **47,** 3239–3245.

BASLER, K., EDLUND, T., JESSEL, T.M. & YAMADA, T. (1993). Control of cell pattern in the neural tube: regulation of cell differentiation by dorsalin-1, a novel TGF-$\beta$ family member. *Cell*, **73,** 687–702.

BRESSLER, J.P., GROTENDORST, G.R., LEVITOV, V. & HJELMLAND, L.M. (1985). Chemotaxis of rat brain astrocytes to platelet derived growth factor. *Brain Res.*, **344,** 249–254.

CHERESH, D.A. (1991). Structure, function and biological properties of integrin $\alpha_v\beta_3$ on human melanoma cells. *Cancer Metast. Rev.*, **10,** 3–10.

CONSTAM, D.B., PHILIPP, J., MALIPIERO, U.V., TEN-DIJKE, P., SCHASHNER, M. & FONTANA, A. (1992). Differential expression of transforming growth factor-$\beta$1, -$\beta$2 and -$\beta$3 by glioblastoma cells, astrocytes and microglia. *J. Immunol.*, **148,** 1404–1410.

DE MARTIN, R., HAENDLER, B., HOFER-WARBINEK, R., GAUGITSCH, H., WRANN, M., SCHLUSENER, H., SEIFERT, J.M., BODMER, S., FONTANA, A. & HOFER, E. (1987). Complementary DNA for human glioblastoma-derived T cell suppressor factor, a novel member of the transforming growth factor-beta gene family. *EMBO J.*, **6,** 3672–3677.

ITO, S., HOSHINO, T., SHIBUYA, M., PRADOS, M.D., EDWARDS, M.S.B. & DAVIS, R.L. (1992). Proliferative characteristics of juvenile pilocytic astrocytomas determined by bromodeoxyuridine labelling. *Neurosurgery*, **31,** 413–419.

IWASAKI, K., ROGERS, L.R., BARNETT, G.H., ESTES, M.L. & BARNA, B.P. (1993). Effect of recombinant tumour necrosis factor-$\alpha$ on three dimensional growth, morphology, and invasiveness of human glioblastoma cells *in vitro*. *J. Neurosurg.*, **78,** 952–958.

KNOTT, J.C.A., EDWARDS, A.J., GULLAN, R.W., CLARKE, T.M. & PILKINGTON, G.J. (1990). A human glioma cell line retaining expression of GFAP and gangliosides, recognized by A2B5 and LB1 antibodies, after prolonged passage. *Neuropath. Appl. Neurobiol.*, **16,** 489–500.

LABOURDETTE, F., JANET, T., LAENG, P., PERRAUD, F., LAWRENCE, D. & PETTMANN, B. (1990). Transforming growth factor type $\beta$1 modulates the effects of basic fibroblast growth factor on growth and phenotypic expression of rat astroblasts in vitro. *J. Cell Physiol.*, **144,** 473–484.

LINDHOLM, D., CASTREN, E., KIEFER, R., ZAFRA, F. & THOENEN, H. (1992). Transforming growth factor $\beta$1 in the rat brain: increase after injury and inhibition of astrocyte proliferation. *J. Cell Biol.*, **117,** 395–400.

MASSAGUÉ, J. (1984). Type $\beta$ transforming growth factor from feline sarcoma virus-transformed rat cells. Isolation and biological properties. *J. Biol. Chem.*, **259,** 9756–9761.

MASSAGUÉ, J. (1990). The transforming growth factor-$\beta$ family. *Annu. Rev. Cell Biol.*, **6,** 597–641.

MAZEWSKI, C., BLISARD, K., BALLARD, E., BLAIR, P., QADIR, K., BOKHARI, S., RAZA, A. & LAMPKIN, B. (1993). Prognostic significance of immunohistochemical detection of transforming growth factor beta (TGF-$\beta$) in central nervous system (CNS) neoplasia (abstract). Growth Control in Central Nervous System International Workshop, Boston, Massachusetts April 30–May 1.

MCKINNON, R.D., PIRAS, G., IDA Jr, J.A. & DUBOIS-DALQ, M. (1993). A role of TGF-$\beta$ in oligodendrocyte differentiation. *J. Cell Biol.*, **121,** 1397–1407.

ROBERTS, A.B., ANZANO, M.A., LAMB, L.C., SMITH, J.M. & SPORN, M.B. (1981). New class of transforming growth factors potentiated by epidermal growth factor. *Proc. Natl Acad. Sci. USA*, **78,** 5339–5343.

RUSSELL, D.S. & RUBINSTEIN, L.J. (1989). *Pathology of Tumours of the Nervous System*, 5th edn, pp. 83–289. Williams & Wilkins: Baltimore.

SAKAI, Y., RAWSON, C., LINDBURG, K. & BARNES, D. (1990). Serum and transforming growth factor $\beta$ regulate glial fibrillary acidic protein in serum-free-derived mouse embryo cells. *Proc. Natl Acad. Sci. USA*, **87,** 8378–8382.

SHEPHERD, G.M. (1983). *Neurobiology*, pp. 164–183. Oxford University Press: New York.

SPORN, R. & ROBERTS, A. (1988). Peptide growth factors are multifunctional. *Nature*, **322,** 217–219.

SPORN, R. & ROBERTS, A. (1992). Transforming growth factor $\beta$: recent progress and new challenges. *J. Cell Biol.*, **119,** 1017–1021.

WILCOX, J.N. & DERYNCK, R.J. (1988*a*). Localization of cells synthesizing TGF-$\alpha$ mRNA in the mouse brain. *Neuroscience*, **8,** 1901–1904.

WILCOX, J.N. & DERNYCK, R. (1988*b*). Developmental expression of transforming growth factors alpha and beta in mouse fetus. *Mol. Cell Biol.*, **8,** 3415–3422.